# Wide-Field Motion Integration in Fly VS Cells: Insights from an Inverse Approach

**DOI:** 10.1371/journal.pcbi.1000932

**Published:** 2010-09-30

**Authors:** Benjamin Torben-Nielsen, Klaus M. Stiefel

**Affiliations:** Theoretical and Experimental Neurobiology Unit, Okinawa Institute of Science and Technology, Onna-Son, Okinawa, Japan; University College London, United Kingdom

## Abstract

Fly lobula plate tangential cells are known to perform wide-field motion integration. It is assumed that the shape of these neurons, and in particular the shape of the subclass of VS cells, is responsible for this type of computation. We employed an inverse approach to investigate the morphology-function relationship underlying wide-field motion integration in VS cells. In the inverse approach detailed, model neurons are optimized to perform a predefined computation: here, wide-field motion integration. We embedded the model neurons to be optimized in a biologically plausible model of fly motion detection to provide realistic inputs, and subsequently optimized model neuron with and without active conductances (g_Na_, g_K_, g_K(Na)_) along their dendrites to perform this computation. We found that both passive and active optimized model neurons perform well as wide-field motion integrators. In addition, all optimized morphologies share the same blueprint as real VS cells. In addition, we also found a recurring blueprint for the distribution of g_K_ and g_Na_ in the active models. Moreover, we demonstrate how this morphology and distribution of conductances contribute to wide-field motion integration. As such, by using the inverse approach we can predict the still unknown distribution of g_K_ and g_Na_ and their role in motion integration in VS cells.

## Author Summary

It is well established that neuronal morphology influences the computation performed by a single neuron. However, it remains largely unknown how these computations emerge from the interaction between dendritic morphology, the distribution of ion-channels and synaptic inputs. To investigate this neuronal morphology-function relationship we employ an inverse approach in which detailed model neurons are optimized to perform a predefined computation. In this work, we set to investigate how dendritic morphology contributes to wide-field motion integration in fly lobula plate tangential cells (LPTCs), cells of which the morphology is assumed to be linked to their function as wide-field motion integrators. The resulting optimized models perform well and share crucial features of LPTC morphology. By analysis of the optimized models, we revealed a match between morphological structures and physiological mechanisms required to perform wide-field motion integration, i.e., we explicitly show the morphology-function relationship in LPTC neurons. Moreover, the optimized distribution of ionic conductances gives rise to predictions about the distribution and role of these conductances in the real neurons. Finally, our findings provide an explanation of dendritic morphologies in terms of the computation they should perform.

## Introduction

Neurons in different animals and brain regions feature a wealth of different dendritic morphologies and distributions of ionic conductances [1], [2]. While the physiological effects of these morphologies and conductance distributions are increasingly understood, how the computational functions these dendrites perform emerge from their morphologies and physiologies is still incompletely known. Computational function is defined here as input-output transformation, resulting from the physiology and subserving the biological purpose of that neuron. Notable exceptions to this incomplete understanding are neurons close the sensory input for which the electrophysiological dynamics are recorded during sensory stimulation, the morphology is known and both can be correlated to the neuron's sensory coding. One such example are the fly lobula plate tangential cells (LPTCs), which responds to visual motion in preferred directions, and which are demonstrated to be wide-field motion detectors [3], [4].

We have recently developed an ‘inverse approach’ to elucidate dendritic structure-function relationships. The underlying assumption of the inverse is that dendritic structure parallels the computational function performed in dendrites. In the inverse approach, we start with a computational function of interest and optimize model neurons including dendritic morphology to perform this function. Here, we apply the inverse approach to investigate the neuronal morphology-function relationship in the fly LTPCs. We focus on a particular type of LPTC, the VS cells that respond to vertical motion. Briefly, VS cells receive motion sensitive signals from the medulla in a retinotopic organization on their dendrites. These inputs are ‘noisy’ insofar they are corrupted by spatial modulation reflecting the activity of individual inputs to the VS cell [3]. While the membrane potential of VS cells at the sites of synaptic input reproduces the fast input dynamics, by the time the signal reaches the axon the cells' physiology and anatomy gets rid of the temporal modulations imposed by their presynaptic local motion detectors. The output of the LPTCs is a smooth signal that encodes the direction (and velocity) of the presented moving stimulus [3], [5]. Thus the function of a single VS cell is *temporal smoothing of motion sensitive inputs to produce a smooth output signal*. This computation is rewarded in the optimization performed in this work. In line with the argument described in [3] we assume that the dendritic morphology computes temporal smoothing. The aim of this study is to identify the morphological building blocks required to perform wide-field motion integration, and to investigate how physiological processes interact with the morphology to perform wide-field motion integration.

In this work, we start by formalizing the notion of temporal smoothing and subsequently optimize different model neurons to perform this computation. We found that our optimized model neurons share crucial morphological features with real VS cell morphologies and that the intrinsic dynamics show remarkable similarity to the real cells. Our results provide an alternative line of support for the hypothesis that the VS cell's morphology contributes to the computation of wide-field motion integration in these cells. Moreover, from our simulations we were able to identify the morphological building blocks required to perform wide-field motion integration and conclude that (i) passive dendrites alone can account for temporal smoothing, and (ii) active ion-channels can be used to balance the (amplitude of the) responses to visual stimulation in the preferred and null-direction. Due to the similarity of our optimized models and real VS cells we can predict the actual -but still unknown- distribution of three ionic conductances and their role in wide-field integration in VS cells. We discuss the significance of our results and compare our findings to a related approach.

## Results

### Generation and optimization of neuron models

**Figure 1:**
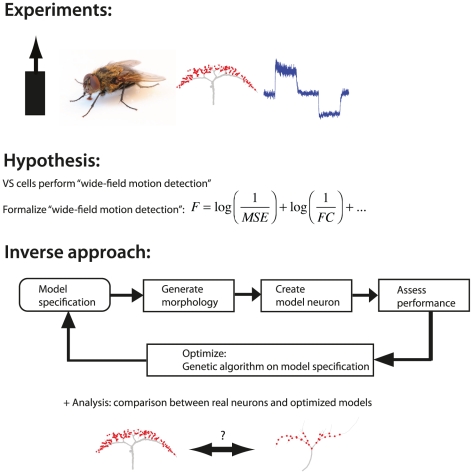
Inverse approach applied to wide-field motion integration. The morphology and physiology of VS cells are well described. We formulate a hypothesis based on the biological data and express this hypothesis in a formula, i.e., the fitness function. Subsequently, we generate model neurons (from a model specification) and assess their performance according to the fitness function. Based on the assessment, the model specification is optimized using a genetic algorithm until models are found that perform the desired function. Afterwards, these optimized model neurons are analyzed for matches with real VS cells. Matches support the notion that the hypothesized function is indeed carried out by these neurons. Details in the methods. (Fly photograph by M. Turney, Wikimedia Commons, VS cell morphology after [5] and voltage data by H. Cuntz.)

The general outline of the type of study performed here is given in figure 1. We start by picking a computational function, in this case a computation generally believed to be a function of VS cells, namely wide-field motion integration. Then we translate the chosen computation into a mathematical expression that quantifies the ability to perform the computation. Subsequently, we optimize model neurons performing the desired computation. In a final step, we analyze the morphology and the underlying physiological mechanisms and compare these findings to real neurons. We refer to this methodology as the inverse approach.

More specifically, we use an algorithm to generate a dendritic morphology from a model specification, i.e., a parameter set consisting of real-numbered values. After generating a morphology, electrical properties and synapses are inserted to obtain a model neuron which is then simulated in the simulation engine neuron [6]. Then a fitness value expressing the ability to perform the predetermined function is computed for each model according to the fitness function.

A genetic algorithm (GA) is used as optimization procedure. It simultaneously and incrementally optimizes a population of model neurons based on the fitness values of the individual model neurons [7]. In GAs, an initially random population of model specifications is optimized by a principle related to ‘survival of the fittest’. As a result, after an increasing number of iterations of the GA, the ‘surviving’ model neurons perform increasingly better at the desired computation.

The crucial step in the GA as adopted here is the correct assessment of the performance of a model neuron as a wide-field motion integrator. We devised a fitness function formalizing important characteristics of the output signals of the VS cells when involved in motion-integration. These characteristics are chosen as follows: wide-field motion integration is the process that temporally smoothens the inputs (1) by decreasing the variance of the membrane potential in the axon close to the connection to the dendritic tree (the somas of fly LPTCs is located outside the neuropil), (2) by increasing the frequency of the output with respect to the input signal, and (3) results in depolarizing and hyperpolarizing membrane potentials upon visual stimulation in the preferred- and null direction, respectively. The fitness function is defined as:
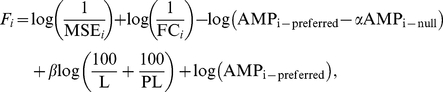
(1)where MSE stands for the mean squared error with respect to the average membrane potential and FC stands for the Fourier component at the input frequency (i.e., the power of the input frequency). The third term evaluates the requirement to respond to stimulation in preferred- and null direction stimulation by rating the difference between the appropriate EPSP amplitudes. We straightforwardly demand equal balance (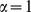) between the amplitudes (AMP) of the membrane shift upon stimulation by motion in the preferred direction and null direction, respectively. While the actual balance in VS cells might be different, this assumption is a good starting point. The fourth term with the total length (L) and average path length (PL) biases for smaller but plausible morphologies [8]. The fifth term avoids trivial solutions due to very small EPSP amplitudes. Because VS cells respond nonspecifically to input-frequency of 1–10 Hz movement [9], [10], we average the performance over three biologically relevant inputs frequencies (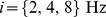). Several heuristics are used to direct the optimization procedure to successfully performing models. A detailed motivation for this fitness function is given in the Methods.

We optimized two types of model neurons: a passive model neuron and an active model neuron which includes specific voltage-dependent conductances [5], [11]. The axon of the active model contains uniformly distributed active conductances similar to the description in [5]. We performed 10 optimization runs (with different random number generator seeds) for both model types to avoid bias originating from the model-generation and model-optimization algorithms. A fitness value of 10 indicates success in wide-field motion integration (see Methods). Additionally, we re-implemented the model described in [5] as a reference for our results (see Text S1).

### Performance as wide-field motion integrators

**Figure 2:**
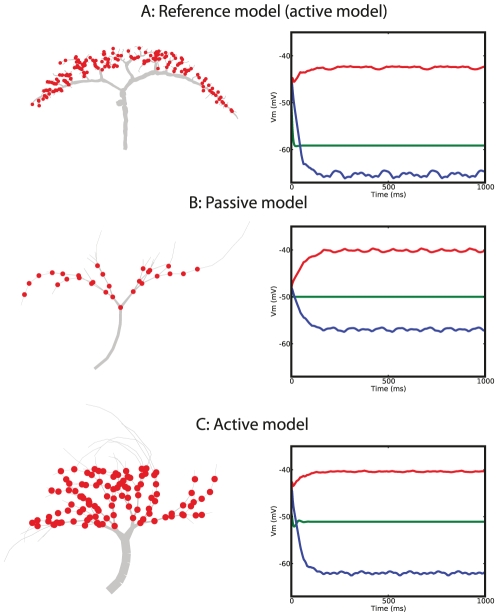
Summary of the results. The rows correspond to the different models: (A) shows the reference model while the best optimized passive and active model neurons are shown in (B) and (C), respectively. The left column illustrates the dendritic tree of the model neuron and its synapses (red dots). The right column shows the membrane potential at the beginning of the axon in rest (green) and upon stimulation in preferred-direction (red) and null-direction (blue). The distinctive characteristics as evaluated in the fitness function such as the variance of the signal and the amplitude of the membrane shift can be observed from these traces.

Both passive and active model neurons were successfully optimized to perform wide-field motion integration. The performance was 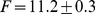 for passive models and 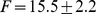 for active model neurons. We also assessed the performance of the reference model and found 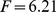; which is a slight underestimate of the performance because the synapses are distributed in the same way as in the optimized models; without taking the kidney-shape of the real VS cell in consideration. However, the larger size and the imprecise balance between the response in preferred- and null-direction evidently lower the fitness value. Thus, according to our fitness function, both active and passive models can perform wide-field motion integration while active model neurons perform better than passive model neurons. The morphology and an illustration of the resulting dynamics in the model neurons are shown in figure 2. The rows represent (from top to bottom) the reference model, the passive model, and the active model. The red dots in the dendrites represent the location of synaptic inputs. The traces in the dynamics panels denote the membrane potential at the start of the axon (i.e, output signal) without visual stimulation (green), or when stimulating with preferred-direction (red) or null-direction movement (blue). The different characteristics that result in the overall performance (such as smoothness and amplitude of the membrane shifts) can be visually inspected in figure 2 (second and third row, right column). These plots show the membrane potential after stimulation with 2 Hz movement; 4 Hz and 8 Hz movement results in similar membrane potential profiles. It can be seen that a depolarizing shift occurs after motion is presented in the preferred direction, and a hyperpolarizing shift after null-direction movement. The amplitudes of these shifts in membrane potential are of almost identical amplitude. Furthermore, the output signal is smooth and does not comprise of the low-frequency oscillations contained in the input signal (see Methods). Together with the formal assessment, these observations demonstrate the ability of the optimized neurons to act as wide-field motion integrators.

**Figure 3:**
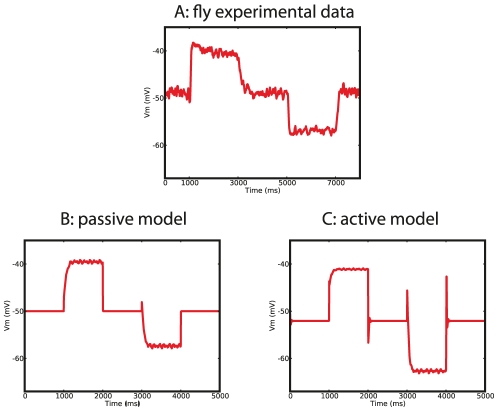
Comparison of optimized performance to real VS cell performance. Response of a real VS cell (A) and two optimized model neurons (B, C) to a similar stimulation protocol is shown. The protocol consists of no motion, preferred-direction motion and null-direction motion. The responses of the models neurons and the real VS cell are similar. We did not directly optimize for this similarity and the high resemblance is an emergent property corroborating the hypothesis of VS performing wide-field motion integration. (Voltage data in A by H. Cuntz.)

The performance of the optimized neurons and the reference neuron are qualitatively similar. Both reference model and optimized neurons have a (1) depolarizing membrane shift with preferred direction stimulation, (2) hyperpolarizing membrane shift with null-direction stimulation, (3) smooth membrane potential which is not a copy of the input signal but is a higher harmonic of the input. Thus, when stimulating the optimized models with a stimulation protocol as used in biological experiments, the membrane response in rest and after stimulation in opposing directions are in agreement with biological data recorded from a VS cell (figure 3). The spike-like event at the onset of the stimulation (with movement in either direction) is an artifact of the implementation of the input signal to the model, i.e. the excitatory and inhibitory synapses provide input to the cell at the same moment but the excitatory synapses have a stronger driving force which result in a brief depolarization. Hence, at the onset of stimulation, a brief depolarization is seen. Additionally, we observed that the ratio between the depolarizing and hyperpolarizing shift in the active model neurons is close to 1 (figures 2C and 3C) and in agreement with experimental data shown in figure 3 (A). The optimized passive model neurons, however, failed to achieve the demanded equality in amplitudes (figures 2B and 3B) which resulted in a lower fitness value. We address this phenomenon later.

### Morphology

All of the successful optimization runs found similar morphologies that enter the target zones to receive synaptic inputs. The blueprint is that a single neuronal branch grows straight towards the target zones and starts branching in the vicinity of the target zones. We stress that this is not a general blueprint of neurons as, for instance, the number of initial segments, and the location of the bifurcations with respect to the root of the dendritic tree differ significantly in different cell types [12], [13]. In the found blueprint, without exception, dendrites enter the target zones from the side of the axon and minimize the size of the dendritic tree. The optimized model neurons have a length of 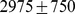 and 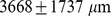 for the passive and active model, respectively. The optimized neurons tend to be smaller than real VS cells as they have fewer short branches and because the model configuration in which synapses are located at roughly 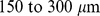 (for comparison, the neuron in figure 2 is 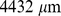). The active models are slightly larger than the passive models due to the phenomenon that active conductances can counteract and/or reinforce passive properties and therefore computations become less dependent on the morphology [7]. Moreover, in a fitness function containing multiple terms, an increase in one term may come at the cost of a small decrease in another term: a large increase in the performance combined with a minor ‘decrease’ of the morphology can result in an increase in the overall fitness.

At this moment two issues arise: is the found blueprint similar to the typical shape of VS cells, and, does the performance as motion integrator depend on this shape?

**Figure 4:**
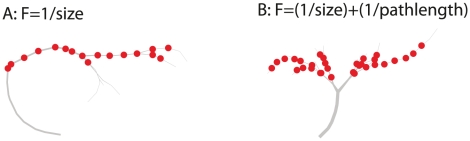
Different morphologies when optimizing for wiring-constraints only. We optimized morphologies *without* the terms for wide-field motion integration, once with only the overall size as constraint and once with the overall size and average path length as constraint. The morphology with the lowest overall size (A) and combined size and path length (B) are shown. The morphology in A deviates substantially from the morphologies found originally, while the morphology in B shares the same blueprint as originally optimized models and VS cells. Both neurons have a significantly lower performance as wide-field motion integrators than the originally optimized passive model neurons.

First, we argue that the blueprint shares crucial features with real VS cells: a single dendritic branch bifurcating strongly before receiving inputs on thin, short dendritic segments where different inputs (from separate small-field motion detectors) arrive at distinct dendritic segments. In the optimization we do not impose explicit constraints on the resulting morphology: only the target zones and the root of the dendritic branch are specified while the number of bifurcations, end-points and size are emergent. Despite the biologically plausible constraints on the target zones [11], we do not model the exact kidney-shape of the lobular plate. Hence, it is evident that there is no strict statistical similarity between our model neurons and VS cells. Equally important but less obvious is the fact that we did not found exceptions to this blueprint despite the possibility of generating radically different morphologies (see below and figure 4). Therefore, we argue that a common morphological blueprint underlies our model neurons which resembles the typical shape of VS cells.

Second, to test the significance of the morphological blueprint for wide-field motion integration we optimized model neurons in the same experimental setup for size (and path length) *without* optimizing for performance; we refer to this type of model as ‘size-only models’. Any difference between the results with and without performance as an optimization constraint clearly indicates a role of function in shaping the neuronal morphology. Figure 4 illustrates the best morphology optimized for wiring length only (A) and for both wiring length and path length (B), respectively. While the morphology in B has the same blueprint as the originally optimized model neurons, the morphology in A has a radically different morphology: it grows a single long branch that passes the target zones horizontally. It is important to note that we did not find any morphology that deviated from the found blueprint when optimizing for function as well. Moreover, when testing the performance of these ‘size-only models’ as wide-field motion integrators (with only passive membrane dynamics), we found that the fitness value was only 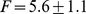 and 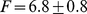 for the models optimized to minimize total size and both size and path length, respectively, where the performance of the passive models was 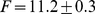. Although a small decrease is to be expected because in the ‘size-only models’ only the morphology was optimized while the conductances were inserted as in [5] to assess their performance. Because of the qualitative difference in the shape, and the nevertheless steep drop in performance when optimizing for wiring constraints only, we conclude that the blueprint found originally (as in figure 2) does contribute to the performance of wide-field motion integration.

### Distribution of active conductances

**Figure 5:**
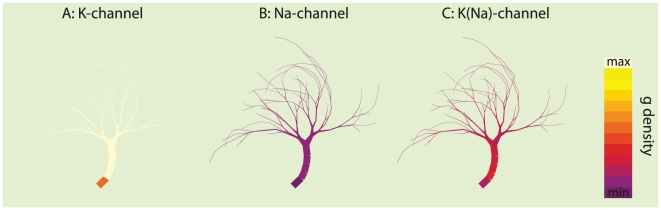
Required conductance distributions to perform wide-field motion integration. All successfully optimized active model neurons showed the same blueprint for the conductance distributions, namely a high density of 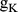 (A) and a low density of 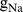 (B) in the dendrites. The distribution of 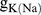 (C) is less constrained and more variable (over different optimized models) because the lack of 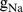 channels. The colors are scaled to the minimum and maximum density allowed in the optimization, 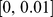, 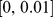 and 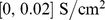 for 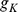, 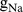 and 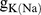, respectively.

In the optimized active neurons, we observed a persistent blueprint for the distribution of active conductances: in all successfully optimized models, a high density of 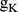 and a low density of 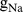 was found in the dendrites. In contrast, 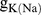 showed more variance as a direct consequence of the lack of sodium currents. The ion-channel distributions of the best optimized active model are illustrated in figure 5. We tested the significance of this blueprint for conductance distributions in two ways. First, we replaced the active conductances in the best performing optimized model by ‘random but plausible’ distributions of conductances, i.e., smooth conductance distributions within the limits of 0–25 times the values described in [5]. We tested 10 such alternative distributions of conductances and found that seven models did not work as wide-field motion integrators anymore, while performance was reduced in the reminding three models. Following the sign-test this decrease in performance is significant (p = 0.05). Second, we removed the active conductances from the successfully optimized active models and observed a performance of only 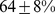 of the original performance resulting in an average performance lower than the optimized passive models. We conclude that the distribution of active conductances as found by our optimizations contributes to wide-field motion integration.

### Role of active conductances

We demonstrated before that the optimized passive models could perform the task of wide-field motion integration. Here, we address the limitation of the passive models, and, the role of active conductances in fly VS cell wide-field motion integration. Despite being an explicit requirement in the fitness function, none of the optimized passive models achieved a balance between the amplitudes of the depolarization and hyperpolarization after preferred- and null-direction motion stimulation, respectively. The reason is the difference in driving force for the excitatory inputs (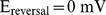) and the inhibitory inputs (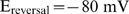). As a result of the 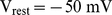, excitatory input results in a larger depolarization than the hyperpolarization resulting from inhibitory inputs.

**Figure 6:**
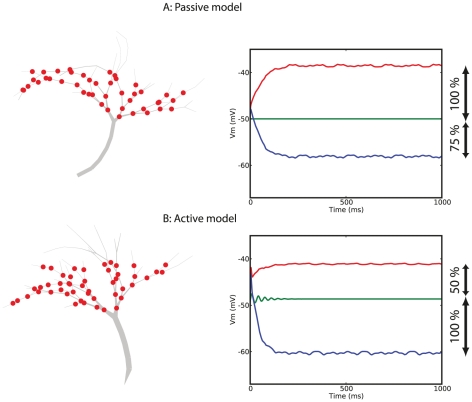
Role of active conductances in balancing depolarization- and hyperpolarization amplitudes. Passive models can only achieve a hyperpolarizing shift of 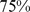 of the depolarization due to different driving forces caused by the synaptic reversal potentials. We optimized passive and active models as before but demanded a balance between the depolarization and hyperpolarization shifts so that the depolarization amplitude had to be 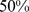 of the hyperpolarization amplitude. None of the optimized passive models exhibited the desired membrane shifts (typical model illustrated in A), while all active models exhibited the demanded membrane shifts (typical model illustrated in B). Therefore, this result follows our hypotheses that active conductances are required in VS cells to achieve biologically realistic balancing between responses in preferred- and null-direction.

Thus, a likely role for the active conductances is a mechanism related to gain control: a balance between the depolarization and hyperpolarization upon stimulation with motion in opposing directions. We tested this hypothesis by optimizing passive and active model neurons to exhibit a hyperpolarization evoked by null-direction motion that has an amplitude of 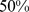 *more* than the depolarization (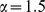 in the fitness function); a requirement that counters the passive dynamics of dendrites because the differential driving force dictates a hyperpolarizing shift of 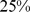 *less* than the depolarizing shift. Figure 6 illustrates the best obtained passive model (A) and active model (B). In accordance to our hypothesis, optimized active models could perform this modified wide-field motion integration while passive model neurons failed as they possess no means to overcome the inequality in the driving force. It is unclear what the exact balancing factor would be in nature because the relative strengths of the excitatory and inhibitory inputs is unknown [14]. Intuitively, the amplitudes should be equal in case they encode not only direction but also velocity. Nevertheless, if the balancing is different from what is expected from the input strength and the driving force, active conductances are required to achieve this balance. Therefore, we conclude that active conductances are required to achieve a (biologically relevant) balance between the depolarizing and hyperpolarizing membrane shifts evoked by motion stimulation in opposing directions.

### Mechanism of computation

**Figure 7:**
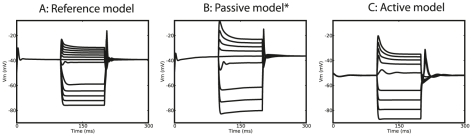
Comparison of the intrinsic dynamics of the reference model and optimized models. Intrinsic physiology is here determined in a current-clamp simulation. Injected currents are (in steps of 1) from 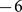 to 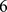 nA in the reference model (A) and from 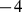 to 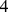 nA in the optimized models (B: passive model, C: active mode). The optimized models show the same qualitative dynamics as the reference model: (i) outward rectification, (ii) a dampening oscillation after 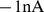 injection, and (iii) strong after-depolarization. Note that the dynamics are not optimized explicitly but emergent, corroborating the hypothesis of wide-field motion integration in VS cells. B: *In the passive model, active conductances were inserted as in the reference model, after optimization with passive properties only.

**Figure 8:**
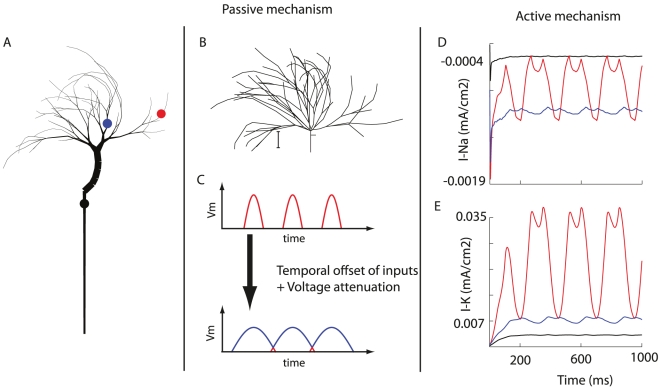
Mechanism of wide-field motion integration by VS cells. Left: full model neuron. Center: Passive mechanism for wide-field motion integration is twofold: electronic distance is similar for each input thus achieving ‘equal weight, equal delay’-summation of temporally shifted inputs in the axon (B), and temporal smoothing by voltage attenuation (C, schematic). Right: Active mechanism that employs Na-currents to boost the smoothed signal in the axon (D) and the strong K-currents in the dendrites which reduce lower the amplitude of individual inputs (E). Color coding of the traces as the locations in (A) indicate.

What is the mechanism underlying wide-field motion integration in VS cells? In order to answer this question we first need to assure that our optimized models are in fact performing wide-field motion integration similarly to VS cells. We already showed that the morphology of the optimized models shares crucial features with VS cell morphologies, and, that the particular distribution of conductances in the optimized models contribute to wide-field motion integration. Both characteristics are emergent as they are not directly rewarded in the fitness function. We also tested a third emergent characteristic dealing with the physiology of the cells directly, namely the electrophysiological dynamics in a current-clamp simulation. To perform the current-clamp simulation of the passive model, we had to insert active conductances into this model in a similar way as described in [5]. The results of this simulation are illustrated in figure 7 and indicate that our models have the same intrinsic physiology as manifested in three features: (1) outward rectification, (2) a dampening oscillation after small injected currents, and (3) strong after-depolarizations. Because of the combination of these three emergent characteristics and their similarity to VS cells we conclude that our optimized neuron model perform wide-field motion integration in a similar way as VS cells do. We identified two physiological components contribute to wide-field motion integration, a passive and an active one (figure 8).

First, the passive mechanism allows all inputs to be electronically equidistant from the axon (figure 8B). As a consequence, the propagation times and attenuation will be similar for each input. By summing all (slightly) temporally offset inputs with equal weights and equal delay in the axon, the signal produced there will ‘connect’ the peaks of all arriving EPSPs. Hence, the output signal skips the gaps between the peaks and is smooth. This process is supported by the dendritic low-pass filtering which widens the EPSPs so that the overlap between EPSP evoked by different inputs widen (figure 8C). It is important to note that the electrotonic distance contributes to a ‘equal weight, equal delay’-summation of the inputs because the neurons have to respond to different motion velocities and does not know on beforehand what the ideal delays (dependent on the velocity) should be. Thus, because of the unknown velocity, the best strategy is to attribute equal weights and delays to each input.

Second, the active physiological mechanism contributes in a complimentary manner to achieve a smooth output signal with a specific membrane shift. The K-conductances, located predominantly at the dendrites, act to cap off the peaks of the input signals signals, thus reducing its amplitude. Since the time when the EPSP peak is reached is offset for inputs from different small-field motion detectors, it is useful for the associated K-conductances to be independently activated. This is achieved due to a certain amount of electrotonic isolation between the regions receiving inputs from different small-field motion detectors, again due to the distinct branching morphology of the VS cells. The Na-conductances, located predominantly at the axon, act to boost the already smoothened signal and regulate the amplitude of the membrane shift.

## Discussion

We used a method for finding optimized neural morphologies and conductance distributions for a chosen computational function. Applying this method to neural wide-field motion integration (temporal smoothing), we found model neurons which performed this task well and resembled fly VS cells in morphology, dynamics and response to inputs. While we cannot be certain that the optimized model neurons perform at, or close to the theoretically achievable optimum for temporal smoothing, we can be fairly confident for two reasons. First, in a previous study using the same inverse-approach but investigating a different task (input order integration), we found that the optimized model neurons are indeed close to the theoretical optimum [7]. Second, the optimized model neurons share crucial features with the real VS cells, the products of evolutionary optimization. As such, our findings further confirm the role of VS cells as wide-field motion integrators.

### Predictions

Based on the demonstrated similarity between the underlying mechanism of wide-field motion integration in model and real VS cells, we can formulate two predictions about the fly VS cells. First, we predict that the actual distributions of the K-channels and Na-channels are similar to the distributions in the optimized model neurons. Thus, we predict that VS cells have a high density of K-channels and few Na-channels in the dendrites, and a relatively high density of Na-channels in the trunk of the dendritic tree and the axon. Second, despite the ability of passive model neurons to perform wide-field motion integration, we predict that the role of the active conductances in real VS cells to achieve a balance between the depolarization and hyperpolarization upon stimulation with preferred-direction motion and null-direction motion, respectively. Although we do not know the exact ratio between depolarization and hyperpolarization amplitudes, the ratio should subserve the biological function of the neurons. The first prediction is testable by immunohistochemical methods, i.e., localizing the ion channel molecules responsible for these currents. The second prediction is testable by pharmacological blockade of the active conductances which should affect the balance between the response amplitudes in these directions.

With these prediction we illustrate an important strength of our inverse-approach to investigate the neuronal morphology-function relationship, the possibility of generating testable predictions about natural systems.

### Morphology-function relationship in VS cells

In a recent study, Cuntz et al. investigated LPTCs and their morphology-function relationship [8]. In this study, they obtained morphologies similar to VS cells based on wiring within an a priori constrained region derived from real VS cell morphologies. Upon establishing statistical identity of the theoretically obtained and real morphologies, they argue that wiring constraints alone suffice to explain the shape of VS cell dendrites. In contrast, we obtained neurons with morphologies and physiologies similar to VS cells based on functional constraints. There are some wiring constraints in our optimization procedure, namely the position of the synaptic zones, but these (potentially) allow for a multitude of neuronal shapes. Thus, without any doubt, functional constraints played a role in determining the morphology of these model VS cells, and we believe that it also did in the evolution of real VS cells. We therefore argue that the dendritic morphologies of VS cells, and of neurons in general, are determined by both wiring and functional constraints.

## Methods

### Two-stage model of optic flow in fly

Optic flow processing in fly is performed in two steps. In the first step small-field motion between adjacent photoreceptors in the retina is detected in the circuits closest to the retina, namely in the lamina and medulla. In the second step all these small-field motion sensitive signals from the whole visual field are integrated in lobula plate tangential cells (LPTCs). The output of the LPTCs is a graded membrane potential shift that encodes the direction (and velocity) of the visual stimulation [3]. In this work, we focus on the LPTC subclass of VS cells that detect vertical motion. We picked this type because it has no connections to other cells in the lobula plate before the actual integration of inputs, i.e., wide-field motion integration is achieved in its totality in this cell alone.

**Figure 9:**
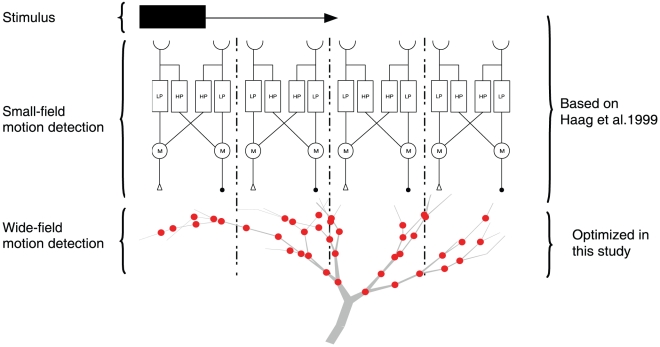
Overview of the model used in this study. The stimulus is external and the subsequent processing is done in two stages. The first stage is small-field motion detection and corresponds to the signal transformation in the lamina and medulla of fly. The second stage corresponds to the wide-field motion integration. In this model, small-field motion detection is performed by Reichardt detectors. The outputs of the small-field motion detectors are projected onto the integrator cell in a biologically accurate way as illustrated. Each small-field motion detector is connected to the integrator cell through an excitatory and inhibitory ribbon synapse that convey graded potentials. Illustrated is a random cell with its connections. The number of detectors shown in the figure is reduced from the actual 20 to 4 for the sake of clarity.

We use a two-stage model of optic flow in fly based on the model by [5]. The first stage is abstract and only functionally mimics the processing of visual stimuli in the circuits before the lobula plate. As in [5], we use Reichardt detectors to perform small-field motion integration. The second stage consist of one multi-compartmental neuron model which is optimized to perform wide-field motion integration. An overview of the model is illustrated in figure 9.

The stimulus in this study is a sine grating with wavelength 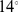 and moving at a velocity of 28, 56 or 112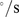, thus realizing a stimulus moving at 2, 4 or 8 Hz.

Our implementation of a Reichardt detector is based on the implementation described in [11]. This implementation uses a delay line (low-pass filter, LPF) and a high-pass filter (HPF) to avoid persistent responses to stationary images. These filters are modeled after [11] and are first-order filters. The low-pass filter has a time constant of 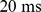 while the high-pass filter has a time-constant of 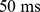. The perceptual range of one such Reichardt detector is based on biological data: The fly eye can perceive a visual field of approximately 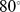 (we make the assumption that the field is equal in all directions) and is locally sampled in an area of approximately 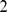 to 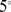 [10], [15]. In the model, visual information is sampled directly in front of one receptor, and the units are located 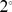 from each other. As two receptors are paired in one detector, 20 detectors cover the complete visual field (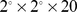).

The inputs to the artificial VS cell are given in fixed locations. More specific, each detector from the first model stage projects on the dendrites of the VS cell in a zone of 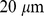 width. When a dendrite passes such a target zone while having a diameter of less than 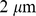, it receives a number of synapses proportional to the dendritic segment length (1 synapse per 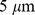). The projections maintain the retinotopic organization of the inputs as in the fly [10], [11]. The synapses are ribbon synapses that convey graded post-synaptic potentials [16]. The precise amount of conductance change in the post-synaptic cell depends on the exact output of the Reichardt detectors.

### Generation and optimization of model neurons

Artificial VS cells are generated algorithmically and optimized to perform wide-field motion integration.

To generate morphologies, we use a *morphogenetic algorithm* that takes a model specification and converts this specification into a model neuron which can be simulated in neuron. Details of the generation process can be found in [7], [17].

A genetic algorithm (GA) is used to optimize the model neurons and operates on the model specification. GAs work in a way inspired by biological evolution and ‘survival of the fittest’. First a population of parameter sets is randomly initiated. Then, each parameter set gives rise to a single model neuron which is subsequently tested for performing the desired function, temporal smoothing of motion sensitive signals. The ‘fittest’ model neurons (the ones with best performance) are allowed to reproduce; essentially mixing the best parameter sets to produce new parameter sets. The new sets are used to generate new model neurons which are in turn tested. By repeating this cycle for a number of times, the population will perform better and better on the desired function and optimization is achieved. The search space is constrained to biologically realistic realms by only allowing a single dendritic tree with minimum and maximum dendritic diameter (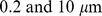) and the maximum conductance densities (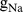: 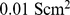, 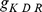: 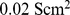, 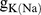: 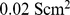).

### Fitness function

Crucial to the success for this approach is a properly defined hypothesis about the computations perform by the neuronal type under investigation. The hypothesis is formalized into a ‘fitness function’ which, in this case, rates the success in performing wide-field motion integration. The fitness function is an integral part of the optimization procedure as it assesses the fitness of a certain model. Here, the underlying hypothesis about VS cell behavior is derived from experimental data and the literature.

The membrane potential trace (averaged over a short window size) of such recordings in shown in figure 3 (top) and demonstrates two basic responses of VS cells to visual motion. First, the output of the VS cell is smooth and does not mimic the fast oscillatory behavior of the inputs. Second, the VS cell responds to both preferred-direction stimulation and null-direction stimulation with either a depolarizing or hyperpolarizing shift in the membrane potential, respectively. These observation are quantified in the fitness function.

In terms of signal processing, we can state that wide-field motion integration (temporal smoothing) is (1) a reduction of the variance and (2) an increase of the frequency of the signal (with respect to the perceived frequency of the inputs). Moreover, for the sake of brevity, we consider the membrane shift upon visual stimulation in the two opposing directions to be of equal amplitude. We can formalize these features of wide-field motion integration as follows. The reduction of the variance is formalized as a reduction of the mean-squared error of the membrane potential. The increase of the frequency of the signal is formalized as a decrease in the power of the frequency dominating the input signal. The elevation of the frequency is also behaviorally important to the fly because it allows for quicker recognition of the stimulus velocity as the time required to observe a full wavelength decreases. According to the Nyquist criterium, the animal needs to monitor the membrane potential for the duration of at least one wavelength to determine the mean of the membrane shift, and thus the velocity of the movement. Shifting to higher frequencies has also been experimentally demonstrated in fly [3]. The relationship between shifts in the hyperpolarizing and depolarizing direction is straightforwardly formalized as a difference between these two voltage shifts.

In addition to these objectives specific to temporal smoothing, we introduced two additional objectives which proved useful in preliminary experiments. The first objective captures a general principle of parsimony in neuronal morphologies: smaller neurons are more desired [18], [19]. The second objective avoids trivial solutions by rewarding for higher amplitudes of the depolarizing membrane shift. Including all the terms explained above, the resulting fitness function is formula 1.

The complete fitness value is only computed according to this fitness formula after the neuron model meet several heuristics: (1) the neuronal morphology should consist of minimally 15 segments, and (2) minimally 1 bifurcation, while (3) the dendrites should connect to at least 15 out of 20 input zones, and (5) the response of the VS cell upon presentation of visual stimuli in the preferred direction should be at least 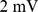. If a model fails to meet all heuristics it receives a penalty proportional to the number of passed heuristics and the ‘distance’ to achieving the next heuristic. The advantage of this type of fitness function is that it provides some cue for badly performing model neurons as how to improve their fitness value in the initial phase of the optimization. Without this feedback, it is often impossible to find good solutions with genetic algorithms [20], [21]. Indeed, these heuristics are not designed to favor a particular morphology (such as the typical VS cell morphology) but only to avoid trivial solutions. In later phases of the optimization, all neurons met all these heuristics, and their performance was assessed by the fitness function alone. With five terms in the fitness function there are many ways to achieve a particular fitness value. However, in preliminary experiments it was found that a fitness of more than 10 is indicative of a good performance as wide-field motion integrator. Because the potential for overfitting to individual terms in the fitness function, we performed a manual check of optimized as well.

Dataset S1 contains the neuron models to reproduce figures 2 and 3.

## Supporting Information

Dataset S1 The passive and active optimized model to reproduce Figures 2 and 3 of the manuscript.
(11.65 MB ZIP)

Text S1 Implementation of the reference VS cell model.
(0.03 MB PDF)
