# Supplementary figures and images for: Wide-Field Motion Integration in Fly VS Cells: Insights from an Inverse Approach
Source: PLoS Comput Biol. 2010 Sep 30;6(9):e1000932. doi: 10.1371/journal.pcbi.1000932 (PMC2947983; doi:10.1371/journal.pcbi.1000932)

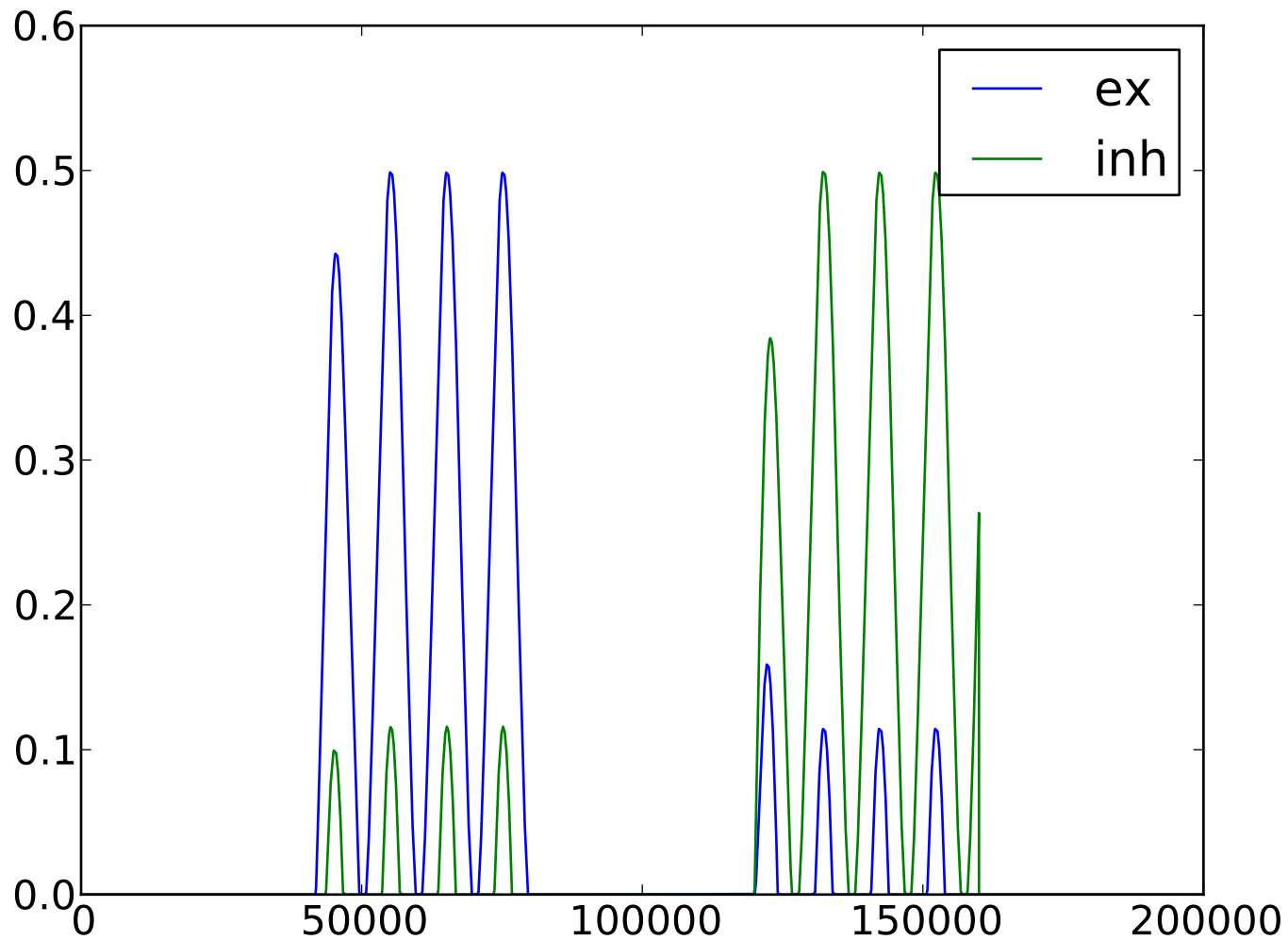

Supplement: Dataset S1 — The passive and active optimized model to reproduce Figures 2 and 3 of the manuscript. (11.65 MB ZIP) [file pcbi.1000932.s001.zip › models/alltrace_inputs.pdf]
