# Supplementary material for: Wide-Field Motion Integration in Fly VS Cells: Insights from an Inverse Approach
Source: PLoS Comput Biol. 2010 Sep 30;6(9):e1000932. doi: 10.1371/journal.pcbi.1000932 (PMC2947983; doi:10.1371/journal.pcbi.1000932)
Supplement: Text S1 — Implementation of the reference VS cell model. (0.03 MB PDF) [file pcbi.1000932.s002.pdf]

VS cell model

The reference VS-cell model -including the channel kinetics- is build after the specifications from [1, 2, 3]. The morphology (72vs4.swc) was downloaded from the NeuroMorpho.org [4] in a standardized format.

The passive electrical properties are identical to the properties listed in Table 1 from [2]. For completeness we repeat these values here: $R_m = 2k\Omega cm^2$, $R_i = 40\Omega cm$, $C_m = 0.8\mu F/cm^2$. Three types of active currents are incorporated in the model: (1) potassium current, (2) sodium current, and (3) sodium dependent potassium current. These currents are described as follows.

$$\begin{aligned} I_{Na} &= (V - R_{rev}) \cdot g_{\max} \cdot m^3 \cdot h \\ I_K &= (V - R_{rev}) \cdot g_{\max} \cdot n^4 \\ I_{K,Na} &= (V - R_{rev}) \cdot g_{\max} \cdot n^4 \end{aligned} \tag{1}$$

The channel dynamics underlying these currents can be described by means of (coupling) ordinary differential equations. Each gating variable used to express the rate of change between open and closed state becomes (m in this example):

$$\frac{dm}{dt} = \alpha_m (V)(1 - m) - \beta_m (V) m. \tag{2}$$

With $\alpha = \frac{m_\infty}{\tau_m}$ and $\beta = \frac{(1-m_\infty)}{\tau_m}$. In [2], m_∞ and β are defined as

$$m_\infty = \frac{1}{1 + e^{\left[\frac{midV - x}{slope}\right]}} \tag{3}$$

, and

$$\tau_m = \frac{\tau_{\max}}{e^{\left[\frac{midVdn - x}{slopedn}\right]} + e^{\left[\frac{midVup - x}{slopeup}\right]}}. \tag{4}$$

The active currents were distributed uniformly over 'the axon and major dendritic branches' [2]. The conductance of the associated channels is as follows: $g_{max} = 3mS/cm2$ for the Na-current, $1mS/cm2$ for the K-current, and $2mS/cm2$ for the K(Na)-current. Other values can be found in Table 1 of [2].

This NEURON implementation is made available through Senselab's modelDB [5] with access number: 116956. Note that this model has $E_{rest} = 0mV$ as in the original publications. For the experiments described here, we lowered all relevant parameters to obtain a realistic resting potential of $-50mV$. However, there are key unresolved differences between the published model and the NEURON implementation as described on the modelDB page.

References

[1] Borst A, Haag J (1996) The intrinsic electrophysiological characteristics of fly lobula plate tangential cells: I passive membrane properties. Journal of Computational Neuroscience 3: 313–336.

[2] Haag J, Theunissen F, Borst A (1997) The intrinsic electrophysiological characteristics of fly lobula plate tangential cells: II Active membrane properties. Journal of Computational Neuroscience 4: 349–369.

[3] Haag J, Vermeulen A, Borst A (1999) The intrinsic electrophysiological characteristics of fly lobula plate tangential cells: III Visual response properties. Journal of Computational Neuroscience 7: 213–234.

[4] Ascoli GA (2006) Mobilizing the base of neuroscience data: the case of neuronal morphologies. Nature Neuroscience Reviews 318(7): 318–324.

[5] Hines M, Morse T, Migliore M, NT C, Shepherd G (2004) ModelDB: A database to support computational neuroscience. Journal of Computational Neuroscience 17: 7–11.
